# Interactions of Circadian Rhythmicity, Stress and Orexigenic Neuropeptide Systems: Implications for Food Intake Control

**DOI:** 10.3389/fnins.2017.00127

**Published:** 2017-03-20

**Authors:** Anna Blasiak, Andrew L. Gundlach, Grzegorz Hess, Marian H. Lewandowski

**Affiliations:** ^1^Department of Neurophysiology and Chronobiology, Institute of Zoology, Jagiellonian UniversityKrakow, Poland; ^2^Neuropeptides Division, The Florey Institute of Neuroscience and Mental HealthParkville, VIC, Australia; ^3^Florey Department of Neuroscience and Mental Health, The University of MelbourneParkville, VIC, Australia; ^4^Institute of Pharmacology, Polish Academy of SciencesKrakow, Poland

**Keywords:** agouti-related peptide, circadian timing system, HPA-axis, neuropeptide Y, melanin-concentrating hormone, orexin, relaxin-3

## Abstract

Many physiological processes fluctuate throughout the day/night and daily fluctuations are observed in brain and peripheral levels of several hormones, neuropeptides and transmitters. In turn, mediators under the “control” of the “master biological clock” reciprocally influence its function. Dysregulation in the rhythmicity of hormone release as well as hormone receptor sensitivity and availability in different tissues, is a common risk-factor for multiple clinical conditions, including psychiatric and metabolic disorders. At the same time circadian rhythms remain in a strong, reciprocal interaction with the hypothalamic-pituitary-adrenal (HPA) axis. Recent findings point to a role of circadian disturbances and excessive stress in the development of obesity and related food consumption and metabolism abnormalities, which constitute a major health problem worldwide. Appetite, food intake and energy balance are under the influence of several brain neuropeptides, including the orexigenic agouti-related peptide, neuropeptide Y, orexin, melanin-concentrating hormone and relaxin-3. Importantly, orexigenic neuropeptide neurons remain under the control of the circadian timing system and are highly sensitive to various stressors, therefore the potential neuronal mechanisms through which disturbances in the daily rhythmicity and stress-related mediator levels contribute to food intake abnormalities rely on reciprocal interactions between these elements.

## Introduction

Circadian (~24 h) fluctuations of different components of the external environment, in particular light-dark conditions, shape animals' physiology and behavior for the optimal synchronization to the cyclical changes encountered. As a consequence of this influence, biological processes called circadian rhythms developed in most organisms (Vaze and Sharma, 2013). In mammals, circadian rhythms are shaped by the master circadian pacemaker, the suprachiasmatic nucleus (SCN), and secondary circadian clocks in brain and peripheral areas (Schibler et al., 2003; Mendoza and Challet, 2009). The SCN coordinates secondary clocks through neural and endocrine pathways, and glucocorticoids released from the adrenal cortex play a key role in the coordination of the circadian timing system (Balsalobre et al., 2000; Barclay et al., 2012). Importantly, mediators of the hypothalamic-pituitary-adrenal (HPA) axis, a key regulator of stress responses, directly and indirectly influence both the circadian timing system and brain centers responsible for food intake control (Dallman et al., 1995; Balsalobre et al., 2000; Segall et al., 2009; Nader et al., 2010).

**Figure 1:**
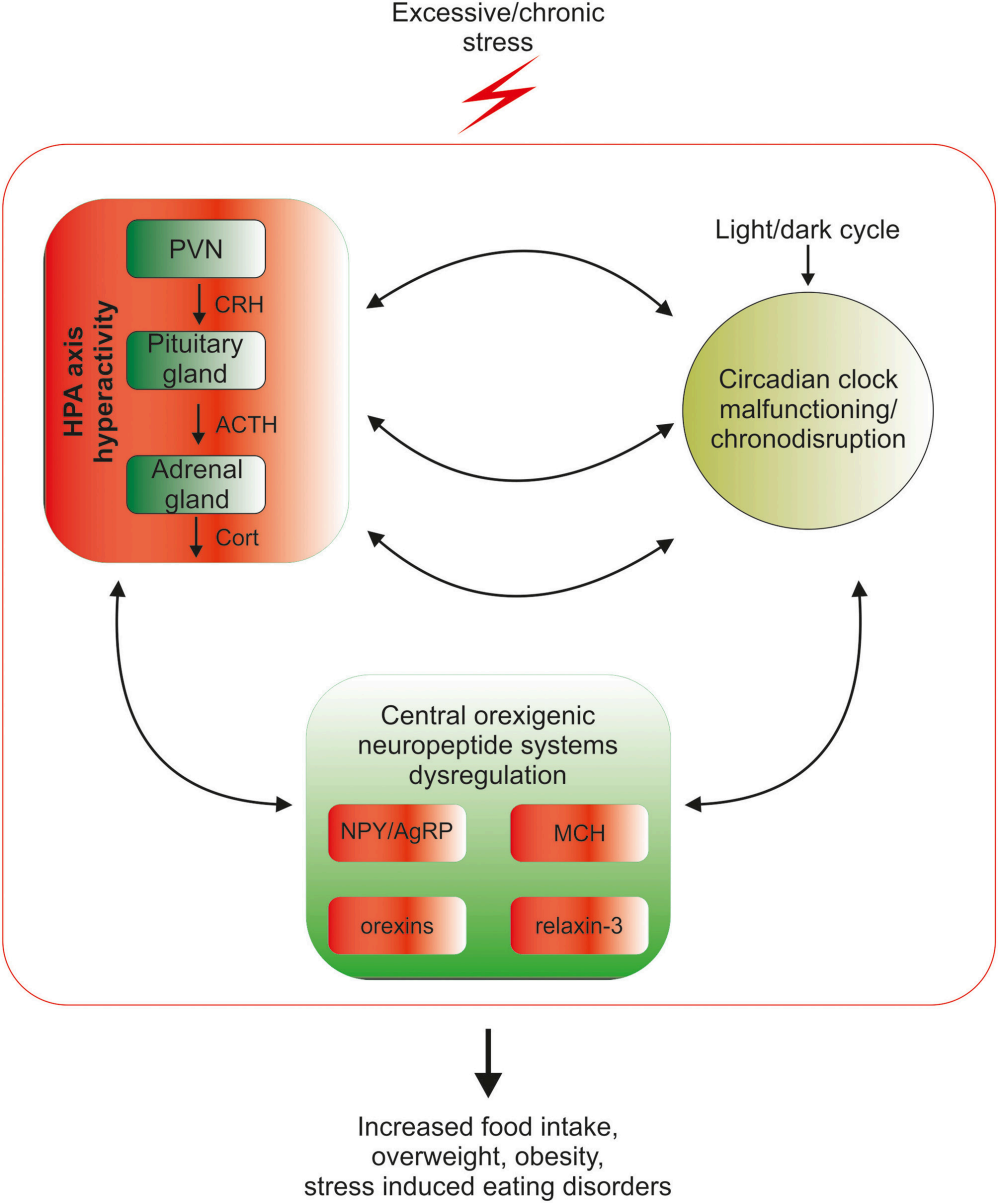
**Mutual relationship between the biological circadian clock, stress and orexigenic peptide systems**. Circadian clock structures in the brain are under direct influence of the stress HPA-axis mediators: corticotropin-releasing hormone (CRH), adrenocorticotropic hormone (ACTH) and glucocorticoids (GCs), therefore excessive exposure to stress disrupts the circadian rhythmicity of the organism. Coincidently, chronic stress through excessive release of HPA mediators deregulates the synthesis and action of orexigenic brain peptides, which can lead to overconsumption of food, weight gain, obesity and stress-related eating disorders. The control of orexigenic peptide synthesis during prolonged stress exposure is dysregulated at several levels, since excessive stress disrupts circadian rhythmicity, which directly controls both neuropeptide release and HPA-axis function. The cycle of excessive stress influences on food intake-promoting peptide system activity and circadian clock structures is closed by the sensitivity of both HPA-axis and clock structures to orexigenic peptide system mediators.

In the industrialized world, chronic exposure to psychological stress, work/activity during late-night hours, and the introduction of artificial light and resultant sleep reduction, all lead to disturbances in the circadian rhythmicity in HPA-axis functioning (Stevens and Zhu, 2015; Koch et al., 2016). At the same time psychological stress and chronodisruption lead to disturbances in food intake-related processes (Kyrou et al., 2006; Antunes et al., 2010). Among other factors, food intake remains under the control of orexinergic neuropeptides, including agouti-related peptide, neuropeptide Y, orexin, melanin-concentrating hormone and relaxin-3, and the goal of this mini-review is to summarize current knowledge about mechanisms linking circadian rhythms, stress and orexigenic neuropeptides, which may underlie stress- and chronodisruption-induced food-intake abnormalities (Figure 1).

## HPA-axis rhythmicity and the circadian timing system—a mutual relationship

The HPA-axis consists of the paraventricular nucleus (PVN), the anterior lobe of the pituitary gland, and the adrenal cortex. PVN neurons synthesize corticotrophin-releasing hormone (CRH) and arginine vasopressin (AVP), which stimulates secretion of adrenocorticotrophic hormone (ACTH) from the anterior pituitary and ACTH controls the release of glucocorticoids (GCs) from the adrenal cortex (Ulrich-Lai and Herman, 2009).

PVN neurons are under direct and indirect control of the SCN (Kalsbeek et al., 1992, 1996). Rhythmic release of neurotransmitters from SCN inputs to the PVN cause circadian oscillations in PVN activity (Tousson and Meissl, 2004) and daily fluctuations in CRH hnRNA and peptide levels (Owens et al., 1990; Girotti et al., 2009). ACTH plasma levels are driven by CRH secretion, but also remain under the control of the SCN (Cascio et al., 1987; Kalsbeek et al., 1996). Similarly GC release is controlled by the circadian master clock through neural control of CRH and ACTH release (Ulrich-Lai and Herman, 2009) and the autonomic innervation of the adrenal gland (Buijs et al., 1999). The rhythmic synthesis of corticosterone depends directly on the master clock, since destruction of the SCN abolishes this rhythmicity (Moore and Eichler, 1972).

Moreover, the adrenal gland has its own clock for circadian GC production (Son et al., 2008) and chronodisruption alters HPA-axis reactivity and plasma GC concentrations (Wu et al., 2008). Not surprisingly, disturbed cyclical functioning of the HPA-axis is implicated in many diseases (Chung et al., 2011). Notably, the activity of the HPA-axis exhibits ultradian rhythmicity and both ACTH and GC secretion is pulsatile in an hourly pattern (Spiga et al., 2011). Similarly to circadian fluctuations, the ultradian pattern of HPA-axis activity is crucial for proper stress responses (Sarabdjitsingh et al., 2010), however, unlike the circadian pattern, ultradian oscillations in GC secretion persist in SCN-lesioned animals and in constant light conditions (Waite et al., 2012). This uncoupling from daily rhythmicity indicates that pulsatile secretion of GC is not SCN-dependent and suggests its reliance on other oscillatory mechanisms.

Importantly, the HPA-axis reciprocally influences the circadian system. Rhythmic GC secretion synchronizes peripheral and central circadian oscillators (Balsalobre et al., 2000; Nader et al., 2010) and is crucial for the synchronization of intrinsic rhythmicity to external factors (Balsalobre et al., 2000). In the majority of tissues GCs affect the expression of clock-related genes, however GCs do not act within the SCN, where the concentration of GC-receptors is low/absent (Balsalobre et al., 2000; Pezuk et al., 2012).

Managing stress involves habituation, which provides appropriate stress coping and avoids harmful, chronic physiological consequences (Spyrka and Hess, 2010). Unfortunately, stressors that are unpredictable and experienced in high intensity lead to disruption of homeostasis, including altered circadian rhythmicity and disturbances in metabolism. One consequence of prolonged stress exposure for humans is overweight and obesity (Coccurello et al., 2009), now a major worldwide public health issue (World Health Organization, 2016). Despite increasing data concerning the reasons for and the mechanisms underlying this phenomenon, much is still to be learnt about the neuronal and humoral underpinnings of stress-induced overconsumption of food.

## Central regulation of food intake: the interaction of orexigenic neuropeptide signaling, stress and circadian rhythmicity

Homeostatic regulation of food intake and energy expenditure relies on central and peripheral signals that are processed within brain centers and peripheral organs. Among many neurotransmitters involved in appetite control, centrally synthetized orexigenic neuropeptides are considered important mediators, and disturbances in their synthesis and/or signaling may contribute to malfunctioning of energy management. In the following sections, data regarding the mutual relationship between major central orexigenic neuropeptides systems, the stress-response axis and circadian systems are provided, indicating potential neuronal mechanisms involved in stress- and chronodisruption-mediated food-intake dysregulation.

### Neuropeptide Y and agouti-related peptide

The arcuate nucleus (ARC) of the hypothalamus is a key appetite regulatory center (Banks, 2010). It contains two major neuronal populations, one synthesizing orexigenic neuropeptide Y (NPY) and agouti-related peptide (AgRP), and a second expressing anorexigenic proopiomelanocortin and cocaine and amphetamine-regulated transcript (Benite-Ribeiro et al., 2016).

Acute and chronic icv NPY administration result in hyperphagia, obesity and changes in metabolism (Beck et al., 1992; Su et al., 2016). Fasting increases ARC levels of NPY and AgRP mRNA (Hahn et al., 1998; Kim et al., 2005) and compromised NPY release delays physiological food intake (Krashes et al., 2013). Central AgRP injections increase feeding (Kim et al., 2000) and fasting increases the expression of AgRP mRNA and peptide (Hahn et al., 1998; Liu et al., 2012). Finally, ablation of AgRP neurons result in acute anorexia (Gropp et al., 2005) and selective activation of AgRP neurons drives feeding behavior (Krashes et al., 2011).

Both NPY and AgRP synthesis are sensitive to HPA-axis mediators. NPY levels increase after exogenous GC treatment (Larsen et al., 1994) and immobilization increases NPY mRNA in the ARC (Conrad and McEwen, 2000). Importantly, prolonged elevations in GC levels lead to overconsumption of food through inhibition of CRH and stimulation of NPY expression (Kaye et al., 1990; Cavagnini et al., 2000).

Interestingly, although AgRP and NPY are co-expressed in the same neuron, their mRNA levels are differentially regulated by stressful events. For example, footshock stress increases NPY mRNA levels, but decreases AgRP mRNA levels (Kas et al., 2005). In rats, repeated footshock (for 14 days) increases ARC AgRP mRNA levels (Helmreich et al., 2005) and chronic (4 weeks) corticosterone treatment increases hypothalamic AgRP, but not NPY, mRNA (Sefton et al., 2016). Psychological stressors, such as restraint stress, reduce the number of AgRP-expressing neurons in the ARC after both acute and repeated exposure (Chagra et al., 2011), highlighting the importance of the duration and type of stressor. Stress hormones may directly act within the ARC, since AgRP-ARC neurons are directly innervated by PVN/CRH neurons, express CRH_1_-receptors and CRH decreases their excitability (Kuperman et al., 2016). Similarly, GC-receptors are highly expressed in the ARC (Morimoto et al., 1996) and their activation stimulates NPY release (Yi et al., 2012) as well as NPY and AgRP gene expression (Shimizu et al., 2008).

At the same time ARC neurons innervate the PVN (Kuperman et al., 2016; Fenselau et al., 2017) and central NPY administration increases the level of CRH, ACTH and corticosterone (Alfalah and Michel, 2004). Moreover, NPY administration into the PVN stimulates food intake (Stanley and Leibowitz, 1985) and induces increased PVN neural activity (Fan et al., 2016); while sustained, viral-mediated overexpression of NPY within this structure results in obesity (Tiesjema et al., 2009). A bidirectional relationship between the HPA-axis and ARC may therefore constitute a positive feedback loop, underlying stress-induced food intake.

Importantly, ARC neuron activity is influenced by the circadian system; indirectly through HPA axis and through direct inputs from the SCN (Yi et al., 2006). Indeed, diurnal rhythm of ARC c-Fos expression (Jamali and Tramu, 1999) and enhancement of AgRP neuron activity during the active phase of the diurnal cycle was demonstrated (Krashes et al., 2013). Simultaneously, NPY/AgRP cells in the vmARC are essential for generating and maintaining circadian rhythms of *ad libitum* feeding (Li et al., 2012); and ARC lesions result in rest-activity disturbances (Wiater et al., 2011). Moreover, ARC NPY/AgRP circuits are crucial for the entrainment of activity by photic cues and entrainment of temperature by food (Wiater et al., 2013). Therefore, NPY/AgRP signaling is a key element of the feeding/energy homeostasis control system, through which stress and chronodisruption may influence food intake.

### Orexins/hypocretins and melanin-concentrating hormone

Orexins/hypocretins are synthesized within the lateral hypothalamus (LH) (Peyron et al., 1998). Orexin neurons are involved in the control of a variety of homeostatic functions including feeding and energy expenditure (Date et al., 1999). When centrally or intraperitoneally injected, orexins stimulate food intake (Edwards et al., 1999). Fasting induces up-regulation of prepro-orexin mRNA levels (Sakurai et al., 1998), and increases the number of excitatory synapses on orexin neurons (Horvath and Gao, 2005). Chemogenetic activation of orexin neurons simultaneously increases locomotor activity and food intake (Inutsuka et al., 2014) and blockade of orexin receptors reduces food intake (Haynes et al., 2000) and binge eating behavior (Piccoli et al., 2012). Orexins are also necessary for arousal maintenance and orexins stimulate arousal-related behaviors (Hagan et al., 1999). A loss of orexin neurons leads to narcolepsy, but does not cause weight loss, and narcoleptic patients suffer abnormalities in energy metabolism and often obesity (Schuld et al., 2000). Similarly, in orexin-deficient mice, in addition to sleep/arousal cycle abnormalities, a lower level of spontaneous physical activity and obesity (regardless of hypophagia) have been described (Hara et al., 2001). Despite an orexigenic effect of acute orexin treatment, chronic icv orexin-A infusion does not lead to weight gain (Yamanaka et al., 1999). Moreover, recent findings reveal that orexin cells activity decreases after eating onset and their silencing leads to eating facilitation (González et al., 2016), which is in line with the obesity observed in orexin-deficient mice (Hara et al., 2001). However, the role of orexins in the maintenance of wakefulness during food searching and reward-related food consumption (Cason et al., 2010) highlights their importance in food intake control.

Orexin neurons are directly activated by CRH and stress (Winsky-Sommerer et al., 2004) and play a key role in stress-induced overconsumption of food (Piccoli et al., 2012), and reinstatement of alcohol- and drug-seeking behavior (Kastman et al., 2016; Schmeichel et al., 2016). The functional link between the HPA-axis and orexin neurons is reciprocal, since orexins evoke an induction of c-*fos* mRNA in the PVN, and an increase in plasma ACTH and corticosterone (Kuru et al., 2000). These pathways may be involved in stress-induced over-activation of orexin neurons, leading to reward-based binge eating behavior.

An accepted role of the orexin system is in the integration of circadian and metabolic influences to shape the arousal and nutritional states of the organism (Selbach and Haas, 2006). Orexins are essential for maintenance of the sleep-wake cycle (Kantor et al., 2009) and strong bidirectional neural connections exist between circadian and orexin systems. Chronodisruption may disturb orexin neuron functioning and disrupted orexin system activity may influence the circadian system. Orexin neurons receive both direct and indirect innervation from the SCN (Abrahamson et al., 2001; Deurveilher and Semba, 2005). Consequently, SCN-dependent circadian patterns are observed in brain orexin levels (Yoshida et al., 2001; Deboer et al., 2004; Zhang et al., 2004) and orexin neurons activation (Marston et al., 2008). On the other hand, SCN neurons are surrounded by orexin fibers and express orexin receptors (Belle et al., 2014). Orexins influence the activity of SCN neurons and are able to induce variable phase shifts in neonatal cultured SCN neurons and phase advances in organotypic brain slices (Klisch et al., 2009). In adult brain, orexins hyperpolarize SCN neurons and enhance the capacity of NPY to shift the phase of *Period1* gene expression in adult brain slices, without an ability to induce such phase shifts alone (Belle et al., 2014). Moreover, orexins modulate the activity of structures involved in non-photic circadian entrainment such as the intergeniculate leaflet (Pekala et al., 2011; Palus et al., 2015) and dorsal raphe nucleus (Kohlmeier et al., 2013).

Within the LH, orexin neurons are intermingled with cells synthesizing melanin-concentrating hormone (MCH) (Broberger et al., 1998). Icv injections of MCH increase food intake in satiated rats (Guesdon et al., 2009), MCH knockout mice are hyperactive and lean (Shimada et al., 1998), and fasting increases levels of MCH mRNA (Bertile et al., 2003). Notably, optogenetic activation of MCH neurons in mice induces sleep, but not food consumption (Konadhode et al., 2013). However, MCH signaling can promote motivational behaviors leading to overconsumption of highly-palatable, calorically-dense food (Georgescu et al., 2005) and is involved in stress-induced binge eating (Pankevich et al., 2010), as well as cocaine (Chung et al., 2009) and alcohol (Duncan et al., 2005; Karlsson et al., 2016a,b) consumption. The involvement of MCH in food intake in animals prompted investigation of potential therapeutic effects of MCH receptor (MCHR) antagonists as anti-obesity agents in humans, but currently no compounds have proceeded to Phase II studies (Macneil, 2013).

MCH synthesis is sensitive to stress, as footshock decreases MCH mRNA levels, an effect mimicked by adrenalectomy and counteracted by dexamethasone replacement (Presse et al., 1992). Chronic mild stress in mice increases MCH-receptor expression in hippocampus (Roy et al., 2007) and repeated restraint up-regulates MCH expression (Kim and Han, 2016). Moreover, treatment of hippocampal neurons with corticosterone *in vitro* increases MCH expression (Kim and Han, 2016). Therefore, MCH neurons constitute another substrate through which stressors can affect feeding behavior (Hervieu, 2003).

Similar to other orexigenic peptide systems, the relationship between MCH signaling and stress is reciprocal. Blockade of MCHR1 has strong anxiolytic and antidepressant effects (Borowsky et al., 2002; Smith et al., 2006) and MCHR1 knockout mice exhibit reduced depressive-like behavior (Roy et al., 2007). Furthermore, the mode of MCH action on the stress axis depends on the circadian time, since significant activation of the HPA-axis through activation of CRH neurons and subsequent stimulation of ACTH release were observed after icv MCH injections in the early light/inactive phase (Jezova et al., 1992). Similarly, icv MCH injections during the light phase lead to increased plasma corticosterone levels and anxiety-like behavior (Smith et al., 2006) and MCHR1 antagonists reverse the effect of chronic and acute stress in mice (Smith et al., 2009; Lee et al., 2011).

In the rat, MCH mRNA expression fluctuates in a circadian manner, with a low level during the light/inactive phase and a peak after the onset of the dark/active period (Bluet-Pajot et al., 1995). MCH is a strong sleep-inducing factor (Verret et al., 2003) and MCH neurons fire during REM sleep, in direct contrast to orexin neurons (Hassani et al., 2009). Direct innervation of the MCH neurons by SCN efferents (Abrahamson et al., 2001) allows for a direct influence of the circadian master clock on MCH levels. The potential feedback of MCH on circadian structures still needs to be verified, but the presence of MCH-receptors on SCN neurons (Chee et al., 2013), and reciprocal connections with neurons influencing circadian clock function, notably orexins neurons (Guan et al., 2002), supports the assumption of a reciprocal interaction of circadian and MCH systems.

The circadian clock control of MCH and orexins synthesis, key neuropeptides involved in stress-induced overconsumption of palatable food and drug-seeking behavior, imposes a need to monitor daily rhythmicity in studies where disturbances in reward-related behaviors are being examined.

### Relaxin-3

The neuropeptide relaxin-3 is synthesized mainly in brainstem *nucleus incertus* (NI) neurons (Ma et al., 2007). Relaxin-3 is highly-conserved, with similarities in distribution between mammalian species, including non-human primate and human brain (Ma et al., 2016), which suggest conservation of its function. Relaxin-3 regulates a variety of physiological processes, including stress responses, motivated behaviors, learning and memory, and food intake (Ma et al., 2016).

An orexigenic effect of relaxin-3 was first described by McGowan et al. (2005) who demonstrated that relaxin-3 was “equipotent” with ghrelin and NPY following icv administration. Further studies confirmed an orexigenic action of relaxin-3 injections (Hida et al., 2006) with higher sensitivity of females to the orexigenic effects of relaxin-3 (Calvez et al., 2015, 2016a). An important site for the orexigenic action of relaxin-3 is the PVN, since intra-PVN injection of the peptide (McGowan et al., 2005, 2006), and virally-mediated secretion of relaxin-3 receptor (RXFP3) agonist within the PVN (Ganella et al., 2013) increases food intake and body weight in rats. The orexigenic action of RXFP3 activation likely involves inhibition of anorexigenic oxytocin (OT) and arginine vasopressin (AVP) synthesis, since a robust reduction in OT and AVP mRNA levels was observed in response to RXFP3 activation in the PVN (Ganella et al., 2013) and relaxin-3 has an inhibitory action on PVN OT and AVP neurons *in vitro* (Kania et al., 2017).

Neurons in the NI are highly sensitive to stress and increases in relaxin-3 mRNA, hnRNA and peptide were observed after both physical (Calvez et al., 2016b) and psychological stressors (Tanaka et al., 2005; Banerjee et al., 2010; Lenglos et al., 2013). Moreover, a majority of relaxin-3 neurons in NI and PAG express CRH_1_-receptors and are excited by CRH injections *in vivo* and *in vitro* (Tanaka et al., 2005; Blasiak et al., 2013; Ma et al., 2013). The relaxin-3/RXFP3 system plays a role in stress-induced binge eating (Lenglos et al., 2013; Calvez et al., 2016b) and stress-induced reinstatement of alcohol seeking (Ryan et al., 2013; Kastman et al., 2016; Walker et al., 2016). Relaxin-3/RXFP3 signaling may reciprocally influence stress responses since icv injections of relaxin-3 in male rats result in increased c-*fos* and CRH mRNA expression in PVN neurons and increased plasma ACTH and corticosterone levels (Watanabe et al., 2011; McGowan et al., 2014; Lenglos et al., 2015). The overall data suggest an important role of relaxin-3 in mechanisms underlying stress influences on food intake related processes.

A growing body of evidence suggests a link between the relaxin-3 system and circadian-related processes and circadian clock structures. Relaxin-3 positive nerve fibers/terminals are present in the main neuronal structures of the circadian system; SCN, IGL and raphe nuclei (Ma et al., 2007) and *in vitro* relaxin-3 receptor activation alters the electrical activity of IGL neurons (Blasiak et al., 2013), which innervate the SCN. Relaxin-3 *and* RXFP3 knockout mice display reduced voluntary running wheel activity during the dark/active phase (Smith et al., 2012; Hosken et al., 2015) and activation of relaxin-3 receptors promotes arousal (Smith et al., 2013). Currently, there are no reports of a circadian rhythmicity in relaxin-3 levels, but sensitivity of relaxin-3 neurons to neuropeptides synthetized in a circadian manner, such as CRH and orexins (Blasiak et al., 2013, 2015) suggest that relaxin-3 expression and/or excitability of NI relaxin-3 neurons may vary over the 24-h cycle.

In conclusion, excessive and prolonged stress experienced on an everyday basis leads to circadian and metabolic disturbances, which constitute overweight and obesity risk factors (Kyrou et al., 2006; Antunes et al., 2010). Considerable experimental evidence indicates that the orexigenic neuropeptides, as critical regulators of energy homeostasis, are important elements of stress- and chronodisruption-induced malfunctioning of feeding behavior. Importantly, the circadian clock, HPA-axis and orexigenic neuropeptide systems display extensive crosstalk, and a better understanding of the mechanisms that control these mutual relationships is necessary for improving treatment strategies for food intake related disorders.

## Author contributions

AB proposed the concept, reviewed the literature, and wrote the manuscript; ALG, GH, and MHL discussed the concept and revised the manuscript critically. All authors accepted the final version of the article.

## Funding

This work was supported by statutory funds of the Institute of Zoology, Jagiellonian University (AB, MHL), by statutory funds of the Institute of Pharmacology, Polish Academy of Sciences (GH) and by National Health and Medical Research Council of Australia Project Grant 1067522 and Research Fellowship 1005985 (ALG).

### Conflict of interest statement

The authors declare that the research was conducted in the absence of any commercial or financial relationships that could be construed as a potential conflict of interest.
